# Maternal excessive gestational weight gain as a risk factor for autism spectrum disorder in offspring: a systematic review

**DOI:** 10.1186/s12884-020-03324-w

**Published:** 2020-10-22

**Authors:** Sorayya Kheirouri, Mohammad Alizadeh

**Affiliations:** 1 Department of Nutrition, Tabriz University of Medical Sciences, Attar Nishabouri St., POBOX: 14711, Tabriz, IR Postal code: 5166614711 Iran; 2 Nutrition Research Center, Tabriz University of Medical Sciences, Tabriz, Iran

**Keywords:** Autism spectrum disorder, Body mass index, Gestational weight gain, Intellectual disability, Genetic susceptibility

## Abstract

**Background:**

Abnormal gestational weight gain (GWG) is a prenatal complication that may contribute to long-term behavioral and neurodevelopmental differences in offspring. This systematic review summarizes research on the association between maternal GWG and risk of autism spectrum disorder (ASD) in offspring.

**Methods:**

Google and electronic databases, including PubMed, SCOPUS, Embase, Cochrane Library and Google Scholar, were searched for original human studies published in English through June 2020. Articles that examined the association between GWG and risk of ASD in offspring were included. Duplicate and irrelevant studies were removed; and data were obtained through critical analysis.

**Results:**

Of 96 articles searched, eight studies were included in the final review. All studies (*n* = 7) investigating the association of maternal excessive GWG with risk of ASD in offspring indicated that high GWG was independently associated with an increased risk of ASD. Of five studies investigating the association of inadequate GWG with the risk of ASD, four indicated that low GWG was not associated with an increased risk of ASD. Of seven studies examining the association of maternal pre-pregnancy BMI or weight with the risk of ASD, five reported that maternal pre-pregnancy BMI or weight did not appear to be independently associated with risk of ASD. The GWG-ASD association is independent of maternal BMI and child’s intellectual disability, but offspring’s genetic susceptibility connection to the GWG-ASD association remains a topic of debate.

**Conclusions:**

The findings suggest that maternal excessive GWG may be associated with increased risk of ASD in offspring. However, insufficient GWG does not appear to have such association.

## Background

Autism spectrum disorder (ASD) is a neurobehavioral disturbance that affects social, communication and behavioral development and can include significant language challenges. According to a 2020 report based on 2016 data from the Centers for Disease Control and Prevention (CDC), ASD prevalence is 1 in 54 children in the United States [1]. The increasing number of people with ASD in recent decades [1] indicates a need for further study of its underlying etiology.

The exact cause of ASD is largely unknown. According to the literature, its etiology is multifactorial and many risk factors, including genetics [2, 3], prenatal and perinatal factors [4, 5], neuroanatomical abnormalities [6, 7], and environmental factors [8] may be involved in the development of ASD.

The prenatal period is a critical period for atypical brain development in people with ASD [9]. Various prenatal complications, such as maternal gestational diabetes [10, 11], advanced parental age [11, 12], bleeding [11], use of prescription medication for the treatment of neuropsychological disorders during pregnancy [13, 14], and meconium in the amniotic fluid [15], have been identified as possible risk factors for ASD during the in-utero period. Abnormal gestational weight gain (GWG) is another prenatal complication which may contribute to long-term behavioral and neurodevelopmental differences in offspring [16–18].

Gestational weight gain is the amount of weight gained by mothers during the period between conception and delivery. Using the mother’s pre-pregnancy body mass index (BMI), acceptable weight gain was classified by the Institute of Medicine (IOM) in 2009. The amount of weight gained during pregnancy influences both maternal and pediatric outcomes and is important to the immediate and the long-term health of both mother and infant. Previous investigations indicated the detrimental influence of undesirable GWG on cognitive development [18, 19]. Excessive GWG has been associated with increased risk of macrosomia [20], neurodevelopmental outcomes and behavioral disorders [21], and prepubertal and postpubertal obesity [22].

Numerous studies have evaluated the association between unfavorable GWG and risk of ASD in children [23–30]. The present systematic review evaluated and summarized current literature in order to determine if a relationship exists between maternal GWG and risk of ASD in offspring.

## Methods

### Search strategy and selection criteria

PRISMA protocol guidelines (2015) were used for this systematic review. A literature search was conducted in the electronic databases of PubMed, SCOPUS, Embase, Cochrane Library and Google Scholar, in addition to Google itself, for all dates up to June 2020 (Table 1). Original articles published in English addressing the relationship between maternal GWG and risk of ASD in offspring were included. Studies on intellectual disability without concurrent ASD diagnosis, neurologic (epilepsy), or other psychiatric diseases (depression, psychosis and anxiety) were excluded. As shown in Table 1, keywords employed for the search were as follows: “gestational weight gain” OR “pregnancy weight gain” OR “weight gain in pregnancy” OR “weight gain during pregnancy” in title-abstract-keywords AND “autism” OR “ASD” OR “developmental delay” OR “developmental disorder” in title-abstract-keywords. The research question was: What is the relationship between maternal GWG and risk of ASD in the child?

**Table 1:** Association between gestational weight gain and risk of autism spectrum disorders: Method of the database search strategy using PubMed, SCOPUS, Google Scholar and Embase

Database (Search conducted up to June 27, 2020)	Search terms^a^	Number of studies searched
PubMed	#1 (((gestational weight gain [Title/Abstract]) OR (pregnancy weight gain [Title/Abstract])) OR (weight gain in pregnancy[Title/Abstract])) OR (weight gain during pregnancy[Title/Abstract])	4323
	#2 (((autism[Title/Abstract]) OR (ASD[Title/Abstract])) OR (developmental delay[Title/Abstract])) OR (developmental disorder[Title/Abstract])	64,806
	#1 combined with #2 ((((gestational weight gain[Title/Abstract]) OR (pregnancy weight gain[Title/Abstract])) OR (weight gain in pregnancy[Title/Abstract])) OR (weight gain during pregnancy[Title/Abstract])) AND ((((autism[Title/Abstract]) OR (ASD[Title/Abstract])) OR (developmental delay[Title/Abstract])) OR (developmental disorder[Title/Abstract]))	15
SCOPUS	(TITLE-ABS-KEY (“gestational weight gain”) OR TITLE-ABS-KEY (“pregnancy weight gain”) OR TITLE-ABS-KEY (“weight gain in pregnancy”) OR TITLE-ABS-KEY (“weight gain during pregnancy”) AND TITLE-ABS-KEY (“autism”) OR TITLE-ABS-KEY (“ASD”) OR TITLE-ABS-KEY (“developmental delay”) OR TITLE-ABS-KEY (“developmental disorder”)) AND DOCTYPE (ar)	24
Google Scholar	allintitle: “autism” OR “ASD” OR “developmental delay” OR “developmental disorder” “weight gain”	24
	with at least one of the words: “autism” OR “ASD” OR “developmental delay” OR “developmental disorder”	
	with the exact phrase: weight gain	
Embase	#1 ‘gestational weight gain’/exp. OR ‘pregnancy weight gain’/exp. OR ‘weight gain in pregnancy’ OR ‘weight gain during pregnancy’	4356
	#2 ‘autism’/exp. OR ‘asd’/exp. OR ‘developmental delay’/exp. OR ‘developmental disorder’/exp.	139,670
	#1 AND #2	28
Cochrane library	#1 (“gestational weight gain”):ti,ab,kw OR (“pregnancy weight gain”):ti,ab,kw OR (“weight gain in pregnancy”):ti,ab,kw OR (“weight gain during pregnancy”):ti,ab,kw	791
	#2 (“autism”):ti,ab,kw OR (“autism spectrum disorder”):ti,ab,kw OR (“ASD”):ti,ab,kw OR (“developmental delay”):ti,ab,kw OR (“developmental disorder”):ti,ab,kw	4481
	#1 AND #2	1
Total		92

^a^Searches were limited to original articles, and studies published in the English language using the appropriate filters and/or search terms depending on the database

### Study screening

Studies retrieved from the search were transferred to an Endnote file and duplicate articles were removed. Titles and abstracts of the remaining articles were assessed by two independent reviewers to screen articles within the scope of this review. Full texts of screened studies were then critically reviewed for eligibility and data extraction. Reference lists of the articles was manually searched to identify additional studies; and when full text of an article was not accessible, it was requested from authors by email. Review articles, animal studies, conference papers, and studies that assessed the relationship of GWG with other health issues such as diabetes or hypertension were excluded. Discrepancies between the two reviewers were resolved through discussion.

### Data extraction

The extracted data included first author and year of publication, country and study design, number of children, age of children, covariates, GWG definition, ASD definition, findings accompanied by odds ratio, confidence intervals or other indicators of correlation, and *p*-value, if available.

### Risk of bias and quality assessment

Selected studies were assessed for methodological quality by two independent reviewers. As shown in supplementary Tables 3 and 4, the Newcastle-Ottawa quality and risk of bias assessment tool for observational cohort and case-control studies [31] was used to evaluate the quality and risk of bias of the included studies based on three domains: selection of exposed and non-exposed groups and ascertainment of exposure; comparability of groups on the basis of the design or analysis controlled for confounders; and outcome regarding assessment and follow-up time. A star system was applied to classify articles as good, fair, or poor quality. Studies with a total score of 6 or higher were considered high quality.

## Results

### Selection of studies

**Fig. 1:**
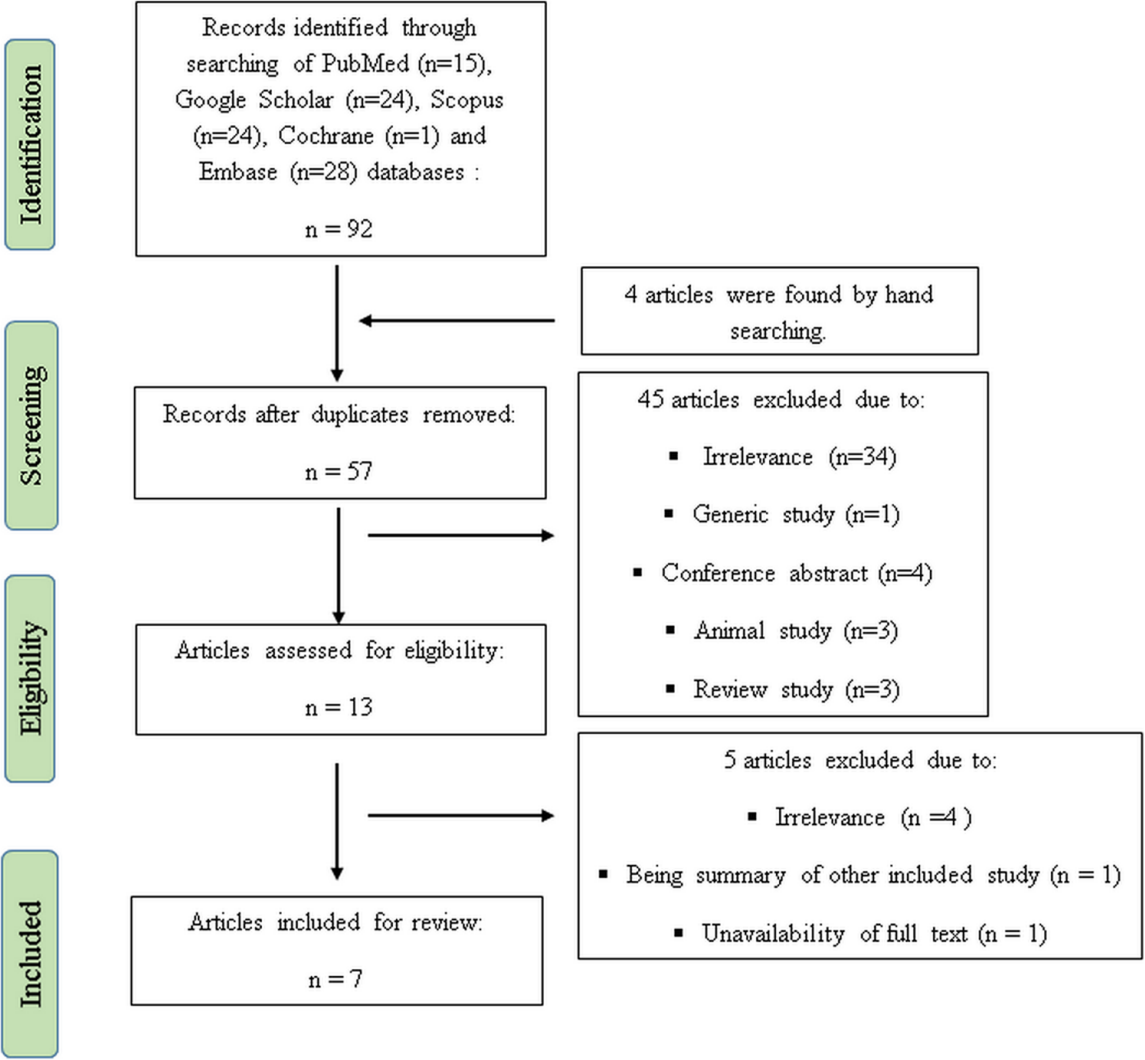
Flow diagram of the study

As shown in Fig. 1, 92 studies were found using the electronic search strategy and four studies were identified through hand searching for a total of 96 that were assessed. Duplicates were deleted, leaving 57 studies. Of those, 13 publications met the topic and scope of the study during screening phase. During the critical review phase, six studies were excluded due to unavailability of full text (*n* = 1), lack of relevance to the study topic (*n* = 4), and a summary of Xiang et al. study [30] (*n* = 1). Hence, a total of seven articles were included in the final review (Fig. 1). The study of Bilder et al. [23] consisted of two different population- and research-based genetic studies, which were considered to be separate studies for the purpose of this review. Therefore, the total count of studies was eight for the final analysis.

### Characteristics of the included studies

Table 2 shows the characteristics of the included studies. All articles were published between 2010 and 2019 and were observational studies (six cohort and three case control). No randomized controlled trial studies were found. All reviewed articles except Dodds et al. study [25] investigated singleton births. Dodds et al. included both singleton and multiple births; however, they adjusted for the effect of multiple births on the relationship between GWG and risk of ASD [25]. All included studies considered the effects of principal confounder factors, including child’s gender, age, birth year, genetic susceptibility, intellectual disability, and mother’s pre-pregnancy BMI, weight, age, education, race/ethnicity, parity, smoking, family income, history of comorbidity, gestational age, as well as father’s age in analyzing the association between maternal GWG and offspring ASD risk. To identify ASD, four of the included studies used ICD (International Statistical Classification of Diseases and Related Health Problems), three studies used DSM-IV-TR (Diagnostic and Statistical Manual of Mental Disorders, fourth edition, text revision), two studies used ADOS-G (Autism Diagnostic Observation Schedule Generic) and two studies used ADI-R (Autism Diagnostic Inventory-Revised) criteria. The definition of excessive or poor GWG varied across the studies; three studies used Institute of Medicine (IOM) guidelines to identify abnormal GWG. Chinese GWG guideline, GWG ≥18 kg as excess and <  7 kg as poor GWG, weight gained < 0.5 kg/week as poor GWG, and continuous model were other utilized methods to define abnormal GWG.

**Table 2:** Characteristics and extracted data of the included studies

Author/ year	Country	Type of study	Number of children/Type of birth	Age of children (year)	Covariates adjusted	GWG definition	ASD definition	Findings OR (95% CI)
Bilder et al./2013 [23]	Utah	ADDM Network cohort (Population-based)	ASD, *n* = 128	8	Intellectual disability, maternal and paternal age and education, parity, pre-pregnancy BMI, gestational age, gender, birth year	Continuous (based on a 5-lb increase)	DSM-IV-TR	Each 5 pounds of weight gained was significantly associated with ASD risk [1.10 (1.03–1.17)].
			Control, *n* = 10,920/Singleton births					
								A 2 SD increase in GWG was associated with risk of ASD (25.00 lb).
								Pre-pregnancy BMI was not associated with ASD.
Bilder et al./2013 [23]	Utah	Genetics Study cohort (research-based)	ASD, *n* = 288	8	Intellectual disability, maternal and paternal age and education, parity, pre-pregnancy BMI, gestational age, gender, birth year	Continuous (based on a 5-lb increase)	ADI-R and ADOS-G	Each 5 pounds of weight gained was significantly associated with ASD risk [1.17 (1.01–1.35)].
			Unaffected siblings, *n* = 493/Singleton births					
								A 2 SD increase in GWG was associated with risk of ASD (24.74 lb).
								Pre-pregnancy BMI was not associated with ASD.
Burstyn et al./2010 [24]	Canada	Cohort	218,890/Singleton births	4–10	Maternal age, weight, pre-pregnancy and gestational diabetes, bleeding, smoking, parity and socio-economic status, child’s birth year, gestational age, gender	Poor GWG: < 0.5 kg/week (26–36 weeks)	ICD-9	Poor weight gain (26–36 weeks, < 0.5 kg/week) was not associated with increased risk of ASD [0.95 (0.57–1.59)].
								Low maternal pre-pregnancy weight (< 45 kg) was associated with increased risk of ASD [RR: 2.15 (1.20–3.85)].
								Maternal weight > 91 kg was not associated with increased risk of ASD [1.18 (0.96–1.44)].
Dodds et al./2011 [25]	Canada	Retrospective, longitudinal Cohort	Total births:	1–17	Year of birth, genetic susceptibility (sibling with ASD, maternal psychiatric disorders, maternal neurological illness), major CNS anomaly, breastfeeding at discharge, infant sex, type of labour, maternal conditions (pulmonary disease, heart disease, renal disease and anemia), pre-pregnancy weight, gestational age.	Excess GWG: ≥18 kg	ICD-9 or ICD-10 codes	GWG ≥ 18 kg in total sample [RR: 1.19 (1.02–1.39)] and in mothers of children with low genetic susceptibility [1.21 (1.03–1.43)] was associated with increased risk of autism.
			*n* = 129,733					
			Autism: *n* = 924/(adjusted for multiple births)			Poor GWG: < 7 kg		
								Inadequate GWG (< 7 kg) was not associated with the risk of autism.
								Pre-pregnancy weight ≥ 90 kg in total sample [1.58 (1.26–1.98)] and in mothers of children with low genetic susceptibility [1.69 (1.34–2.14)] was associated with increased risk of autism.
Gardner et al./2015 [26]	Sweden	Prospective cohort	Total children:	≥ 4	Maternal BMI, gestational age, infant sex, birth year, parity, maternal age, paternal age, maternal country of birth, socioeconomic status, parental psychiatric history, genetic susceptibility, intellectual disability.	Based on IOM guidelines	ICD-9, ICD-10, and DSM-IV codes	Both insufficient [1.17 (1.04–1.31)] and excessive GWG [1.12 (1.01–1.25)] were independently associated with ASD.
			*n* = 333,057					
			ASD: 6420/Singleton births					
								Every 2.3 kg (5 lb) increase in GWG was associated with increased risk of autism [1.03 (1.00–1.06)].
								Matched sibling analyses showed elevated risk of ASD with excessive GWG [1.48 (0.93–2.38)].
								Maternal overweight/obesity was associated with increased risk of offspring ASD [25 ≤ BMI < 30: 1.31, (1.21–1.41); BMI ≥ 30: 1.94 (1.72–2.17)].
Shen et al./2018 [28]	China	Case-control	Autism: *n* = 705	2–9	Child’s gender and age, parental age and family income	Based on Chinese GWG guidelines	DSM-IV-TR criteria	Excessive GWG was associated with autism risk in the entire sample [(1.327, 1.021–1.725)].
			Control: *n* = 2236/Singleton births					
								Excessive GWG increased the risk of autism in overweight/obese mothers [2.468 (1.102–5.526)].
								Inadequate GWG was not associated with the risk of autism.
								Maternal pre-pregnancy BMI might not be independently associated with the risk of autism.
Windham et al./2019 [29]	USA	Case-control	ASD: *n* = 540	2–5	Pre-pregnancy BMI, maternal age, education, race/ethnicity, parity, smoking, intellectual disability, child sex	Based on IOM guidelines	SCQ score ≥ 11, ADI-R, ADOS	Maternal total GWG was higher in the ASD group than other groups.
		SEED	Developmental delays: *n* = 720					
			Control: n = 776/Singleton births					Continuous model: 1.06 (1.02–1.10)
								Exceeds IOM/ACOG recommendation: 1.29 (1.00–1.66)
								Exceeding clinically recommended GWG was significantly higher (51%) in ASD group than other groups.
								Associations of ASD with higher GWG were stronger in quintiles 4 and 5.
								Quintile 4 (35– ≤ 44 pounds): 1.52 (1.05–2.22)
								Quintile 5 (≥44 pounds): 1.58 (1.08–2.31)
								In the continuous model, each 5-pound increase of GWG was associated with6% increased odds of ASD.
								Insufficient GWG was not associated with risk of ASD.
								Maternal pre-pregnancy BMI was not associated with ASD.
Xiang et al./2015 [30]	USA	Retrospective longitudinal cohort	322,323 children	A median of 5.5 years after birth	Birth year, maternal age, parity, gestational age, education, maternal race/ethnicity, household income, history of comorbidity (≥1 diagnosis of heart, lung, kidney, or liver disease; cancer), child sex	Continuous (per 4 kg)	ICD-9 codes 299.x or equivalent KPSC codes	GWG (per 4 kg) [HR: 1.67 (1.10–2.53)] and maternal pre-pregnancy BMI were modestly and positively associated with ASD risk.
			ASD: *n* = 3388/Singleton births					

*ADDM* Autism and Developmental Disabilities Monitoring (ADDM); *ASD* autism spectrum disorders; *BMI* body mass index; *CI* confidence interval; *IOM* Institute of Medicine; *GWG* gestational weight gain; *HR* Hazard Ratio; *OR* Odds Ratio; *SEED* the Study to Explore Early Development; *RR* Relative Risk; *ICD* International Statistical Classification of Diseases and Related Health Problems, 9th revision; *DSM-IV-TR* Diagnostic and Statistical Manual of Mental Disorders, fourth edition, text revision; *ADOS-G* Autism Diagnostic Observation Schedule-Generic; *ADI-R*Autism Diagnostic Inventory-Revised

### Quality of articles

All included studies were rated as good quality (Tables 3 and 4, Supplementary). The quality scores of all six cohort studies were 9, where 9 represents the lowest degree of bias (Table 3, Supplementary). The quality scores of two case-control studies ranged from 8 to 9 (Table 4, Supplementary).

### Maternal excessive GWG and risk of ASD in offspring

As shown in Table 2, seven studies investigated the association of maternal high GWG with risk of ASD in offspring. All seven studies indicated that high GWG was significantly associated with increased risk of ASD. Dodds et al. [25], in a retrospective longitudinal cohort study over 129,733 births, diagnosed 924 children with ASD after 1 to 17 years follow up from birth, and found that GWG ≥ 18 kg was significantly correlated with an increased risk of ASD. Gardner et al. [26], in a cohort study with 333,057 children ≥4 years, of whom 6420 were identified with ASD, reported that excessive GWG independently correlated with risk of ASD. In a case-control study, Shen et al. [28] found that greater GWG was associated with risk of ASD in the whole sample, also enhanced the risk of ASD in overweight/obese mothers. Windham et al. [29], also in a case-control study (ASD: *n* = 540, control: *n* = 776, aged 2 to 5 years), reported that excessive GWG was associated with risk of ASD, and, further, each 5-pound increase in GWG was correlated with 6% increased odds of ASD. Similar findings were also reported by two other studies (Table 2) [23, 30]. Bilder et al. [23] in a population-based cohort study (ASD: *n* = 128, control: *n* = 10,920) and in a genetic research-based study (ASD: *n* = 288, unaffected siblings: *n* = 493) on singleton births found that each 5 pounds of weight gained was significantly associated with increased risk of ASD. Xiang et al. [30] in a retrospective longitudinal cohort study on 322,323 singleton births (ASD: *n* = 3388) reported that each4 kg increase in GWG was positively associated with increased risk of ASD.

### Maternal insufficient GWG and risk of ASD in offspring

As shown in Table 2, five studies investigated the association of inadequate GWG with risk of ASD in offspring. Four indicated that insufficient GWG was not associated with increased risk of ASD [24, 25, 28, 29]. Burstyn et al. [24], in a cohort study over 218,890 births, after 4–10 years follow up, reported that poor weight gain (26–36 weeks, < 0.5 kg/week) was not associated with increased risk of ASD. Dodds et al. [25], in a cohort study (total children: *n* = 129,733; ASD: *n* = 924; ages 1–17 years) found that inadequate GWG (< 7 kg) was not associated with increased risk of ASD. Shen et al. [28], in a case-control study (control: *n* = 2236; ASD: *n* = 705; ages 2–9 years) found that inadequate GWG (based on a Chinese recommendation) was not associated with increased risk of ASD. Windham et al. [29], in a case-control study (control: *n* = 776; ASD: *n* = 540; ages 2–5 years) reported that insufficient GWG (based on IOM recommendation) was not associated with increased risk of ASD.

However, Gardner et al. [26], in a cohort study (total children: 333,057; ASD: 6420; ages ≥4 years) reported that insufficient GWG (based on IOM recommendation) was independently associated with increased risk of ASD.

### The role of child’s genetic susceptibility and intellectual disability in the GWG-ASD relationship

Two of the included studies considered the confounding effect of the child’s genetic susceptibility on the GWG-ASD relationship. One study reported that the significant relationship between GWG and ASD was independent of genetic susceptibility [25], while the other reported that the association may be influenced by the confounder factor [26].

Three reviewed studies addressed the role of child’s intellectual disability in the GWG-ASD association [23, 26, 29]. All showed that excessive GWG increased the odds of ASD in ASD children with or without intellectual disability.

### The role of maternal pre-pregnancy BMI in GWG-ASD relationship

Of the included studies, seven studies examined the association of maternal pre-pregnancy BMI (*n* = 5) or weight (*n* = 2) with risk of ASD in offspring (Table 2). Five studies found that maternal pre-pregnancy BMI and weight ≥ 90 kg were not associated with increased risk of ASD in offspring [23 (population-based study and research-based study),24,28,29]. Bilder et al. [23] reported that maternal pre-pregnancy continuous BMI was not associated with ASD risk and concluded that GWG-ASD association was independent of pre-pregnancy BMI. Burstyn et al. [24] found that maternal pre-pregnancy weight ≥ 90 kg was not associated with increased risk of ASD in offspring. Shen et al. [28] showed that maternal pre-pregnancy low (< 18.5 kg/m2) and high (> 24 kg/m2) BMI were not significantly associated with risk of ASD. The author concluded that the maternal pre-pregnancy BMI might not be independently associated with ASD risk. Windham et al. [29] reported that maternal pre-pregnancy BMI (in all categories; < 18.5, 25.0–29.9 and ≥ 30 kg/m2) was not associated with risk of ASD.

However, two studies reported contrary findings [25, 30]. Dodds et al. [25] showed that maternal pre-pregnancy high weight (≥ 90 kg) was associated with increased risk of ASD. Xiang et al. [30] found that maternal pre-pregnancy continuous BMI and BMI ≥30were positively associated with ASD risk.

Nevertheless, in all studies that assessed the GWG-ASD relationship, the effect of maternal pre-pregnancy BMI or weight were considered and the observed link between GWG and ASD was independent.

## Discussion

The findings of the present review showed that excessive maternal GWG is associated with higher risk of ASD in offspring. In this regard, it is important to pay attention to the following three pivotal points.

### Child’s genetic susceptibility connection to the association between maternal GWG and offspring ASD

In this respect, only two studies considered this point with two contrasting findings. Dodds et al. [25] reported that a significant relationship between GWG and ASD was independent of the child’s genetic susceptibility, since the association remained significant when the analyses were restricted to children with high genetic susceptibility (having an autistic sibling or a mother with a psychiatric or neurologic disease). The result suggests that the observed association is unlikely to be attributable to familial confounding. Gardner et al., in a large study in Sweden [26], performed matched sibling analyses to compare affected children with their unaffected pairs to assess whether observed correlations between maternal GWG and offspring ASD might be the consequence of confounding by shared familial factors. The analysis showed that the association did not remain significant and this may indicate that the result could be influenced by residual confounding, although the authors did not reach this conclusion and suggested an elevated risk of ASD with excessive GWG in matched sibling analyses. Taken together, child’s genetic susceptibility connection to the GWG-ASD association remains debatable and this area requires further investigation.

### Child’s intellectual disability connection to the association between maternal GWG and offspring ASD

Three of the included studies considered the role of child’s intellectual disability in the association between maternal excessive GWG and offspring ASD. Bilder et al. [23] found that a significant GWG-ASD relationship was independent of the existence of comorbid intellectual disability among individuals with ASD, because the significant association persisted when analyses were limited to ASD children with a normal IQ. Windham et al. [29] found that elevated GWG similarly increased the odds of ASD in ASD children with or without intellectual disability. Similarly, Gardner et al. [26] showed that increased GWG similarly affected the odds of ASD in ASD children with or without intellectual disability across the entire studied population, while the odds of ASD in children without intellectual disability significantly increased in mothers with normal baseline BMI. Taken all together, it appears that the association between maternal excessive GWG and offspring ASD is independent of child’s intellectual disability.

### Maternal BMI-GWG interaction and risk of offspring ASD

Although most studies considered the confounding role of maternal pre-pregnancy BMI in GWG-ASD relationship and indicated that excessive GWG might increase the risk of ASD independent of BMI, however, several studies suggested an interaction between maternal BMI and GWG leading to increased risk of ASD in offspring. Shen et al. [28] found that the risk of ASD significantly rose in mothers with high pre-pregnancy BMI along with greater GWG. The authors concluded that the interaction between maternal BMI and GWG was significantly related to offspring ASD risk. Windham et al. [29] reported that the GWG-ASD association did not change by BMI in a continuous model. However, mothers with high BMI were more likely to have excessive weight gain (~ 55%) than mothers of normal weight (~ 35%) and the relation of ASD to greater GWG was more obvious, although non-significant, in overweight/obese mothers. Nevertheless, Gardner et al. [26] found when the analysis of GWG-ASD association was limited to mothers with a normal pre-pregnancy BMI for differentiating potential impacts of GWG from baseline BMI, the risk pattern did not change compared to the entire sample. The authors suggested that excessive GWG was independent of BMI correlated with ASD. Collectively, it is speculated that the association of GWG-ASD independent of maternal BMI is prevailing; however, further investigations are warranted.

Most of the studies used the WHO categorization of BMI [32]; however, two studies used continuous BMI for analysis and two used pre-pregnancy weight instead of BMI, which limits comparability of the results with respect to the BMI-GWG-ASD association.

### Fetus gender connection to the GWG-ASD association

ASD is a sex-specific neurological condition which affects more males than females. This may indicate that underlying mechanisms of sex differentiation may contribute to the neurobiology of ASD. Sex-specific differences in the occurrence of ASD may be attributable to sex-hormone specific effects. High perinatal testosterone levels have been suggested as a potential risk factor for neurodevelopmental disorders, including ASD [33–37]. In this regard, male fetuses would be more affected since testosterone concentration in the amniotic fluid of male fetuses is significantly higher than those of female fetuses at all stages of gestation [38]. The results of this review showed that high GWG may be a risk factor for ASD. According to some evidence, high GWG is accompanied by high testosterone levels [39]. This may indicate that child neurodevelopment may be associated with GWG in a sex-specific manner. It is suggested that fetal testosterone level to be taken into account in the analyzing of the relationship between GWG and risk of ASD.

### Mechanisms for the relationship between maternal excessive GWG and offspring ASD risk

None of the articles reviewed mechanistically studied the role of GWG on ASD risk and the underlying mechanisms connecting maternal excessive GWG to offspring ASD remain unclear. However, the dysregulation of steroid hormones, leptin, and pro-inflammatory cytokines during gestation have been projected as possible mechanisms involved in psychopathology. Endogenous steroid hormones (for example, testosterone, estrogen, progesterone, or cortisol) released by mother, placenta, and fetal gonads and adrenal glands generate a fetal steroid environment [40]. These steroid hormones, as environmental factors, may influence fetal gene transcription and expression through DNA binding during susceptible stages of embryonic development [40]. Any impairment in the fetal steroid hormone environment can lead to dysfunction in the body or even death. Inappropriate levels of fetal steroid hormones such as increased fetal testosterone levels have been reported to be associated with autistic traits [33–35]. As well, a positive relationship has been reported between increased levels of amniotic steroid hormones and ASD [41]. Lof et al. [42], in a longitudinal study of Swedish women, found that plasma levels of progesterone during pregnancy were positively associated with GWG.

Disturbance of leptin is another suggested mechanism contributing to psychopathology. Leptin has a vital role in fetal growth and development during pregnancy [43]. Any dysregulation in fetal leptin levels leads to mental health impairments and neurodevelopmental disorders, including ASD [44]. High plasma leptin in early childhood has been proposed as a potential biomarker for ASD [45] and increased circulating leptin is consistently observed in individuals with ASD [44, 46, 47]. According to previous investigations, fetal leptin levels are affected by maternal leptin levels [48, 49]. Walsh et al. [49] demonstrated an association between maternal and fetal leptin levels at each time point of pregnancy period. Elevated levels of leptin have also been reported in the placental vascular endothelial cells which are associated with maternal obesity [50]. Variation in the interchange of leptin among mother, placenta, and fetus may influence the development of the fetus and increase the risk of disease in later life [51]. Higher maternal leptin levels have also been shown to be associated with greater GWG [52]. The results of two birth cohort studies found that excessive GWG correlated with greater leptin levels in either cord blood or post-delivery maternal serum [53]. Patro-Małysza et al. [54] also reported greater umbilical cord leptin levels in neonates born to mothers with excessive GWG. Further, Vargas-Aguirre at al [55]. found that umbilical cord blood leptin levels were higher in neonates born to mothers with high GWG. Collectively, it is thought that excessive GWG may likely associate with in-utero leptin disturbance, which in turn may lead to increased risk of ASD. However, further studies are needed to support the association of fetal leptin levels and risk of ASD.

**Fig. 2:**
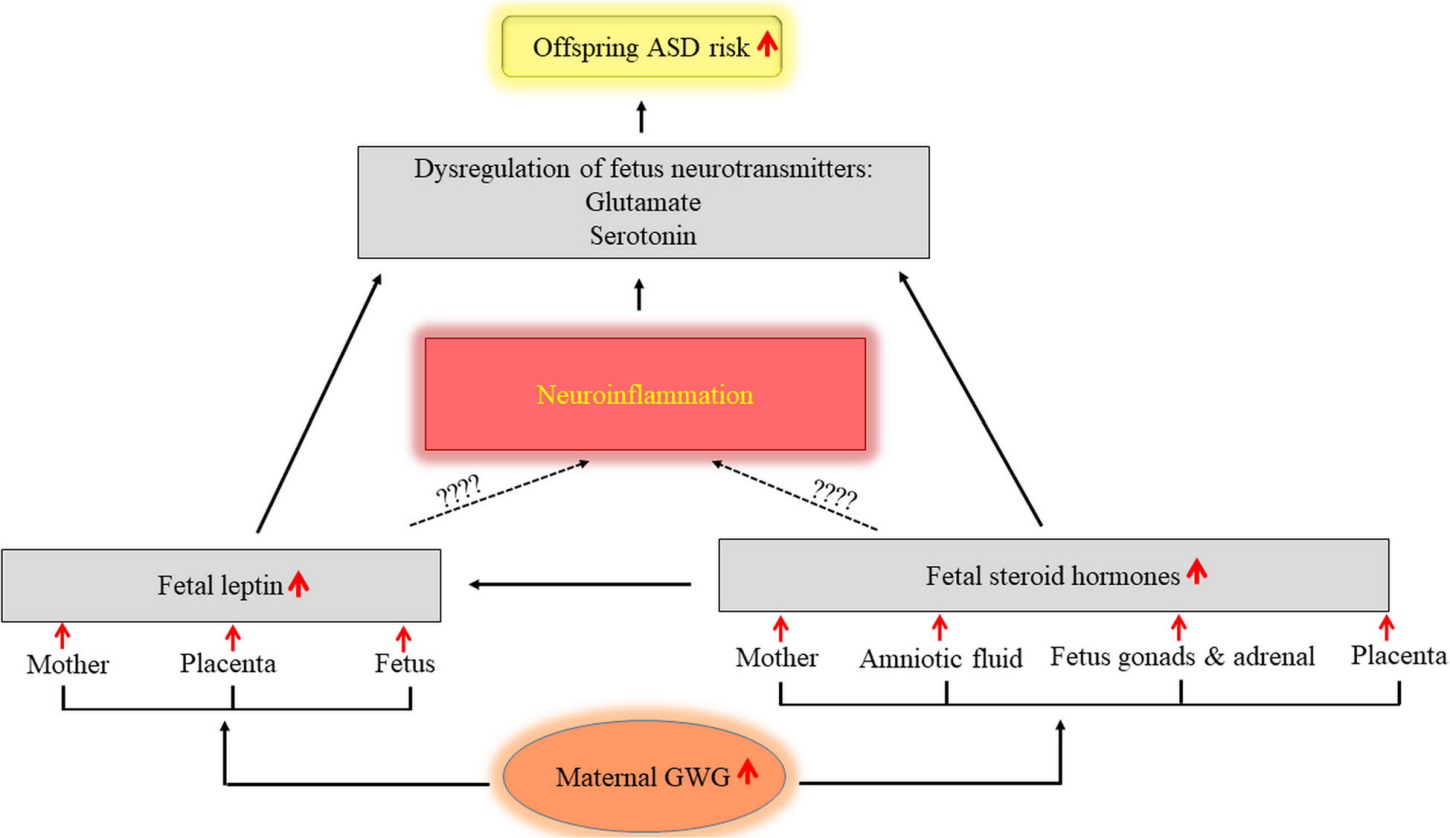
A possible mechanistic model of GWG and ASD relationship. ASD, autism spectrum disorders; GWG, gestational weight gain

Leptin is able to cross the blood-brain barrier and enter the brain. As shown in Fig. 2, the brain leptin-glutamate or leptin-serotonin interactions have been proposed as possible mechanisms involved in the development of neurodevelopmental disorders, including ASD. Serotonin and glutamate are well-known neurotransmitters that have critical roles in neural activities and social behaviors. Clinically, abnormal levels of these neurotransmitters are linked with many neurodevelopmental disorders, including ASD [56, 57]. Serotonin regulates neural development and alterations in its concentration during development can have life-long effects. Exposure to abnormal levels of serotonin during the prenatal period may contribute to behavioral impairments in adulthood [58]. Excessive levels of glutamate have also been reported to have neurotoxic properties [59]. Calapai et al. [60] demonstrated that leptin injections caused a dose-dependent increase of serotonin in mice. Yadav et al. [61] showed that leptin decreased serotonin synthesis and prevented neuronal activity of serotonergic neurons in the ventromedial hypothalamus. Haque and Haleem [62] indicated that serum leptin was inversely associated with circulating serotonin in working men. Fuente-Martín et al. [63] reported that leptin regulated astrocyte-specific glutamate. Further, Yu and Cai [64] showed that excess central leptin increased glutamatergic pathway activation.

Interaction of steroid hormones with the neurotransmitters is another possible pathway for the development of neurodevelopmental disorders (Fig. 2). Zlotnik et al. [65], in a study on men and women, showed that blood glutamate levels were negatively associated with plasma levels of estrogen and progesterone. Raz et al. [66] found that serotonin was regulated by sex steroid hormones and could influence mood. Ladisich [67] found that progesterone injections dose-dependently increased serotonin turnover. According to the evidence, steroid hormones are linked to the regulation of leptin and the leptin receptor [68–70]. Thus, steroid hormones might indirectly contribute to the pathogenesis of ASD through the leptin interface. However, more research is required to confirm the interaction of leptin or steroid hormones with the neurotransmitters involved in occurrence of ASD, in the prenatal period. The role of neuroinflammation in the projected pathways should also be a focus for future study.

## Conclusion

The findings suggest that increased risk of offspring ASD is associated with maternal excessive GWG and is not likely with insufficient GWG. The inclusion of several cohort studies with very large sample sizes (> 300,000 participants), in particular a prospective cohort study, in the current review may possibly enhance the power of these findings. It appears that the association between maternal excessive GWG and offspring ASD is independent of maternal BMI and child intellectual disability. Further investigation is needed to clarify the impact of child’s genetic susceptibility on the association between maternal GWG and offspring ASD. More research is required with regards to interaction between GWG and biochemicals involved in the etiology or pathophysiology of ASD in the prenatal period.

### Application of the findings

This study highlights that pregnancy excessive weight gain, which exhibits the metabolic and nutritional condition of the mother throughout pregnancy, may have long-term neurodevelopmental effects on children and could be employed as a serious predictive biomarker of ASD. Focusing attention on helping women adhere to GWG guidelines is imperative to achieving a healthy weight gain during pregnancy.

### Strengths of the study

Using large sample sizes and consideration of the role of potential confounding factors in the GWG-ASD association in the included studies were strengths of the study.

### Limitations of the study

Various criteria were used to identify ASD and GWG across the studies, which may influence the comparability of the results. None of the studies investigated the role of biochemical markers such as leptin, steroid hormones, neurotransmitters, or neuropeptides involved in the pathogenesis of ASD in the prenatal period to determine underlying mechanisms connecting maternal excessive GWG with offspring ASD.

## Supplementary information

**Additional file 1 Table S3**. Newcastle-Ottawa scale for assessment of quality of six included cohort studies assessing the relationship of gestational weight gain and risk of autism spectrum disorder (each asterisk represents if individual criterion within the subsection was fulfilled)

**Additional file 2 Table S4**. Newcastle-Ottawa scale for assessment of quality of three selected case-control studies assessing the relationship between gestational weight gain and risk of autism spectrum disorders (each asterisk represents if individual criterion within the subsection was fulfilled)

## Data Availability

All data extracted from the studies are included in this published article.

## Abbreviations

ASD: Autism spectrum disorders
BMI: Body mass index
IOM: Institute of Medicine
GWG: Gestational weight gain
