# Supplementary material for: Maternal excessive gestational weight gain as a risk factor for autism spectrum disorder in offspring: a systematic review
Source: BMC Pregnancy Childbirth. 2020 Oct 22;20:645. doi: 10.1186/s12884-020-03324-w (PMC7579946; doi:10.1186/s12884-020-03324-w)
Supplement: Supplementary file 1 — Additional file 1 Table S3. Newcastle-Ottawa scale for assessment of quality of six included cohort studies assessing the relationship of gestational weight gain and risk of autism spectrum disorder (each asterisk represents if individual criterion within the subsection was fulfilled) [file 12884_2020_3324_MOESM1_ESM.docx]

| **Table 3 Supplementary.** Newcastle-Ottawa scale for assessment of quality of six included cohort studies assessing the relationship of gestational weight gain and risk of autism spectrum disorder (each asterisk represents if individual criterion within the subsection was fulfilled) | | | | | | | |
| --- | --- | --- | --- | --- | --- | --- | --- |
| Quality assessment criteria | Acceptable (*) | Bilder et al., 2013 [23] | Bilder et al., 2013 [23] | Burstyn et al., 2010 [24] | Dodds et al., 2011 [25] | Gardner et al., 2015 [26] | Xiang et al., 2015 [30] |
| **Selection** | | | | | | | |
| Representativeness of exposed cohort? | Truly representative |  |  | * | * | * |  |
|  | Somewhat representative | * | * |  |  |  | * |
| Selection of the non-exposed cohort? | Drawn from same community as the exposed cohort | * | * | * | * | * | * |
| Ascertainment of exposure? | Secured records | * | * | * | * | * | * |
|  | Structured interview |  |  |  |  |  |  |
| Demonstration that outcome of interest was not present at start of study? | yes | * | * | * | * | * | * |
| **Comparability** | | | | | | | |
| Comparability of cohorts on the basis of the design or analysis controlled for confounders | The study controls for maternal age and child gender | * | * | * | * | * | * |
|  | Study controls for other factors | * | * | * | * | * | * |
| **Outcome** | | | | | | | |
| Assessment of outcome? | Independent blind assessment |  |  |  |  |  |  |
|  | Record linkage | * | * | * | * | * | * |
| Was follow-up long enough for outcomes to occur | yes | * | * | * | * | * | * |
| Adequacy of follow-up of cohorts | Complete follow up- all subject accounted for | * | * | - | * | * | * |
|  | Subjects lost to follow up unlikely to introduce bias- number lost less than or equal to 20% or description of those lost suggested no different from those followed. | - | - | * | - | - | - |
| **Overall Quality Score (Maximum = 9)** | | 9 | 9 | 9 | 9 | 9 | 9 |
| NS=not stated | | | | | | | |
