# Supplementary material for: Maternal excessive gestational weight gain as a risk factor for autism spectrum disorder in offspring: a systematic review
Source: BMC Pregnancy Childbirth. 2020 Oct 22;20:645. doi: 10.1186/s12884-020-03324-w (PMC7579946; doi:10.1186/s12884-020-03324-w)
Supplement: Supplementary file 2 — Additional file 2 Table S4. Newcastle-Ottawa scale for assessment of quality of three selected case-control studies assessing the relationship between gestational weight gain and risk of autism spectrum disorders (each asterisk represents if individual criterion within the subsection was fulfilled) [file 12884_2020_3324_MOESM2_ESM.docx]

| **Table 4** **Supplementary.** Newcastle-Ottawa scale for assessment of quality of three selected case-control studies assessing the relationship between gestational weight gain and risk of autism spectrum disorders (each asterisk represents if individual criterion within the subsection was fulfilled) | | | |
| --- | --- | --- | --- |
| Quality assessment criteria | Acceptable (*) | Shen et al., 2018 [28] | Windham et al., 2019 [29] |
| **Selection** | | | |
| Is the case definition adequate? | Yes, with independent validation | * | * |
| Representativeness of the cases (how were cases selected) | Consecutive or obviously representative series of cases | * | * |
| Selection of Controls | Community controls | * | * |
|  | Hospital controls |  |  |
| Definition of Controls | Not being ASD | * | * |
| **Comparability** | | | |
| Comparability of cases and controls on the basis of the design or analysis | Study controls for gender | * | * |
|  | Study controls other confounders | * | * |
| **Exposure** | | | |
| Ascertainment of exposure | Secure record (e.g. surgical record/research records) |  | * |
|  | Structured interview where interviewer blind to case/control status | * | * |
|  | Interviewer not blinded to case/control status |  |  |
| Same method of ascertainment for cases and controls | yes | * | * |
| Non-Response rate (dropouts) | Same rate for both groups | - | - |
| **Overall Quality Score (Maximum = 9)** |  | 8 | 9 |
